# Post-surgical Orthodontic Management Using Clear Aligners: A Systematic Review of Stability, Efficiency, and Patient Outcomes

**DOI:** 10.7759/cureus.100967

**Published:** 2026-01-06

**Authors:** Zainab M AlGhafli, Dalal T Mahjoub, Saja A Alzhrani, Abdulelah A Altheyab, Hamed Muhanned H Aljubab, Saeed A Alzahrani, Majed O Bakkar, Najem A Alotaibi, Rawan N Albishry, Dalal Alturaif, Afnan A Bahkali, Laila Alanaz

**Affiliations:** 1 Dentistry, King Faisal University, Riyadh, SAU; 2 Dentistry, King Abdullah Bin Abdulaziz University Hospital, Mecca, SAU; 3 Dentistry, Jazan University, Jizan, SAU; 4 Dentistry, Jouf University, Sakakah, SAU; 5 Dentistry, King Abdulaziz University, Jeddah, SAU; 6 Dentistry, Riyadh Elm University, Riyadh, SAU; 7 General Dentistry, King Abdullah Bin Abdulaziz University Hospital, Mecca, SAU; 8 Dentistry, Vision College, Riyadh, SAU; 9 Restorative Dentistry, Private Clinic, Riyadh, SAU; 10 Dentistry, Heraa Medical Complex, Riyadh, SAU; 11 Dentistry, Private Clinic, Riyadh, SAU

**Keywords:** clear aligner therapy, invisalign, orthognathic surgery, patient-reported outcomes, post-surgical orthodontics, surgery-first approach, treatment efficiency

## Abstract

Clear aligner therapy has gained increasing attention as an alternative to fixed appliances in surgical-orthodontic treatment, particularly during the critical post-surgical phase. This systematic review evaluated the effectiveness of clear aligners in post-surgical orthodontic management, focusing on skeletal and dental stability, treatment efficiency, and patient-centered outcomes. A comprehensive search of PubMed, ScienceDirect, Google Scholar, and the Cochrane Library identified 10 eligible studies involving 258 patients. Owing to substantial heterogeneity in surgical procedures, aligner protocols, outcome measures, and follow-up durations, a meta-analysis was not feasible; therefore, findings were synthesized narratively. Across the included studies, skeletal and dental stability achieved with clear aligners was comparable to outcomes achieved with fixed appliances, with no significant differences in short-term relapse. Several studies reported improved treatment efficiency, including reduced overall treatment duration in surgery-first protocols and fewer clinical appointments during pre-surgical preparation. Patient-centered outcomes consistently favored clear aligners, with randomized trials demonstrating better periodontal health, reduced discomfort, and improved quality of life compared with fixed appliances. Perioperative outcomes were similar between groups, indicating the safety of incorporating aligners into orthognathic workflows. Despite promising results, the evidence remains limited by heterogeneity and a lack of long-term, controlled studies. Clear aligners represent an effective and patient-friendly alternative in post-surgical orthodontic management, offering comparable clinical outcomes with potential advantages in comfort, efficiency, and periodontal health.

## Introduction and background

Orthognathic surgery is a foundational treatment for correcting moderate-to-severe dentofacial skeletal discrepancies that cannot be managed by orthodontic treatment alone, with the primary goals of restoring functional occlusion, facial harmony, and long-term skeletal stability [1]. For over a century, conventional fixed appliances have been the undisputed orthodontic modality of choice throughout the surgical-orthodontic workflow, facilitating precise pre-surgical decompensation and post-surgical finishing [2]. However, the inherent characteristics of fixed appliances - including aesthetic limitations, challenges in maintaining oral hygiene, and patient discomfort - have driven a growing demand for more esthetic and comfortable treatment alternatives.

In response to this demand, clear aligner therapy (CAT), pioneered by systems such as Invisalign®, has transitioned from a solution for minor orthodontic corrections to a viable option for managing complex malocclusions, including those requiring surgical intervention [1]. A recent systematic review found that clear aligners may provide similar dental and skeletal outcomes compared with fixed appliances in the orthognathic setting while offering improved patient satisfaction and periodontal health [3]. The removable nature of clear aligners offers significant potential advantages, such as improved oral hygiene, dietary flexibility, and enhanced treatment comfort, which contribute to greater patient satisfaction [4]. From a patient-centered perspective, randomized clinical evidence indicates that CAT is associated with improved oral health-related quality of life and reduced treatment-related discomfort compared with fixed appliances [5]. The integration of CAT into orthognathic surgery is further propelled by advances in digital dentistry, particularly virtual surgical planning, which allows for the seamless incorporation of orthodontic objectives with osteotomy simulations in a digital workflow [1]. In addition, the use of aligner auxiliaries - such as attachments, elastics, and skeletal anchorage - has been shown to enhance the accuracy and effectiveness of complex tooth movements, as supported by systematic evidence evaluating their biomechanical impact [6].

The application of CAT in orthognathic surgery spans pre-surgical, surgical, and post-surgical phases. In the orthodontics-first approach, pre-surgical orthodontic decompensation is performed to align teeth relative to their basal bone prior to surgery, whereas the surgery-first approach involves performing orthognathic surgery before orthodontic treatment, followed by post-surgical orthodontic finishing [3]. Orthodontic decompensation refers to the correction of dental compensations that mask underlying skeletal discrepancies, enabling accurate surgical correction. Post-surgically, they facilitate occlusal refinement and finishing [7]. Recent reviews and clinical studies have begun to substantiate its role, reporting that CAT can effectively manage various dentofacial deformities, such as skeletal Class II and III malocclusions, with skeletal stability comparable to that achieved with fixed appliances [2]. Furthermore, emerging evidence suggests that CAT may offer benefits in treatment efficiency, including a potential reduction in overall treatment duration, particularly in surgery-first protocols, and a decreased number of required clinical appointments [8]. From a patient-centered perspective, recent randomized controlled trials (RCTs) provide high-quality evidence that patients treated with CAT experience significantly better periodontal health and superior quality-of-life outcomes compared to those in fixed appliances [9].

Despite these promising findings, the current evidence base is characterized by heterogeneity in surgical procedures, aligner protocols, and outcome measures [1]. Much of the existing literature is composed of retrospective cohort studies, case reports, and case series, with a noted scarcity of long-term, high-quality controlled studies. This heterogeneity, coupled with unclear patient selection criteria in some studies, necessitates a cautious interpretation of the results and highlights a significant gap in the literature. Consequently, there is a pressing need to systematically synthesize the current evidence on the stability, efficiency, and patient-reported outcomes of using clear aligners specifically in the post-surgical phase - a critical period for determining final treatment success. Therefore, the objective of this systematic review is to critically evaluate and synthesize the existing evidence on the use of CAT for post-surgical orthodontic management, with a specific focus on skeletal and dental stability, treatment efficiency, and patient-centered outcomes.

## Review

Methodology

Study Design and Registration

This systematic review synthesized current evidence on post-surgical orthodontic management using clear aligners, focusing on stability, treatment efficiency, and patient-reported outcomes. The review followed the Preferred Reporting Items for Systematic Reviews and Meta-Analyses (PRISMA) guidelines [10]. Although the protocol was not registered in PROSPERO (International Prospective Register of Systematic Reviews), all methodological steps were structured to ensure transparency and reproducibility.

Information Sources and Search Strategy

A comprehensive electronic search was conducted in PubMed, ScienceDirect, Google Scholar, and the Cochrane Library from inception to October 2025. The search strategy combined controlled vocabulary and free-text terms related to orthognathic surgery, clear aligners, postoperative orthodontics, and treatment outcomes. Key search terms included “clear aligner”, “Invisalign”, “orthognathic surgery”, “jaw surgery”, “post-surgical orthodontics”, “dentoalveolar surgery”, “stability”, and “treatment efficiency”. The search strategy was customized for each database. No restrictions were applied to study design or publication year, but only articles published in English were considered. Full database-specific search strings are provided in the Supplementary Materials. Additionally, the reference lists of all included studies and relevant reviews were hand-searched to identify additional eligible publications.

Eligibility Criteria

Studies were selected using predefined inclusion and exclusion criteria framed by the PICO model (Table 1).

**Table 1:** PICO framework for the systematic review

PICO Component	Description
Population (P)	Patients of any age and sex who underwent orthognathic or dentoalveolar surgery (single-jaw or bimaxillary) followed by orthodontic treatment
Intervention (I)	Post-surgical orthodontic management using clear aligner therapy (CAT) (e.g., Invisalign or similar systems), applied in surgery-first or orthodontics-first protocols
Comparator (C)	Conventional fixed orthodontic appliances, hybrid protocols (aligners combined with fixed appliances), or no direct comparator where applicable
Outcomes (O)	• Skeletal stability (relapse, cephalometric changes) • Dental and occlusal stability (PAR index, Angle classification) • Treatment efficiency (overall duration, number of visits, refinements) • Patient-reported outcomes (comfort, pain, periodontal health, quality of life, satisfaction) • Perioperative and post-operative complications

The population comprised patients who underwent orthognathic or dentoalveolar surgery followed by orthodontic treatment with clear aligners. Interventions included any postoperative clear-aligner system. Comparators included fixed appliances, hybrid protocols, or no comparator. Outcomes included skeletal and dental stability, treatment duration, efficiency metrics (e.g., refinement needs, number of aligners), patient-reported outcomes (comfort, satisfaction, quality of life), and complications. Eligible study designs included RCTs, controlled clinical trials, cohort studies, case-control studies, cross-sectional studies, and case series with >5 patients. Animal studies, in vitro research, narrative reviews, editorials, letters, surgical technique papers lacking postoperative orthodontic data, and case reports with ≤5 patients were excluded.

Study Selection

**Figure 1:**
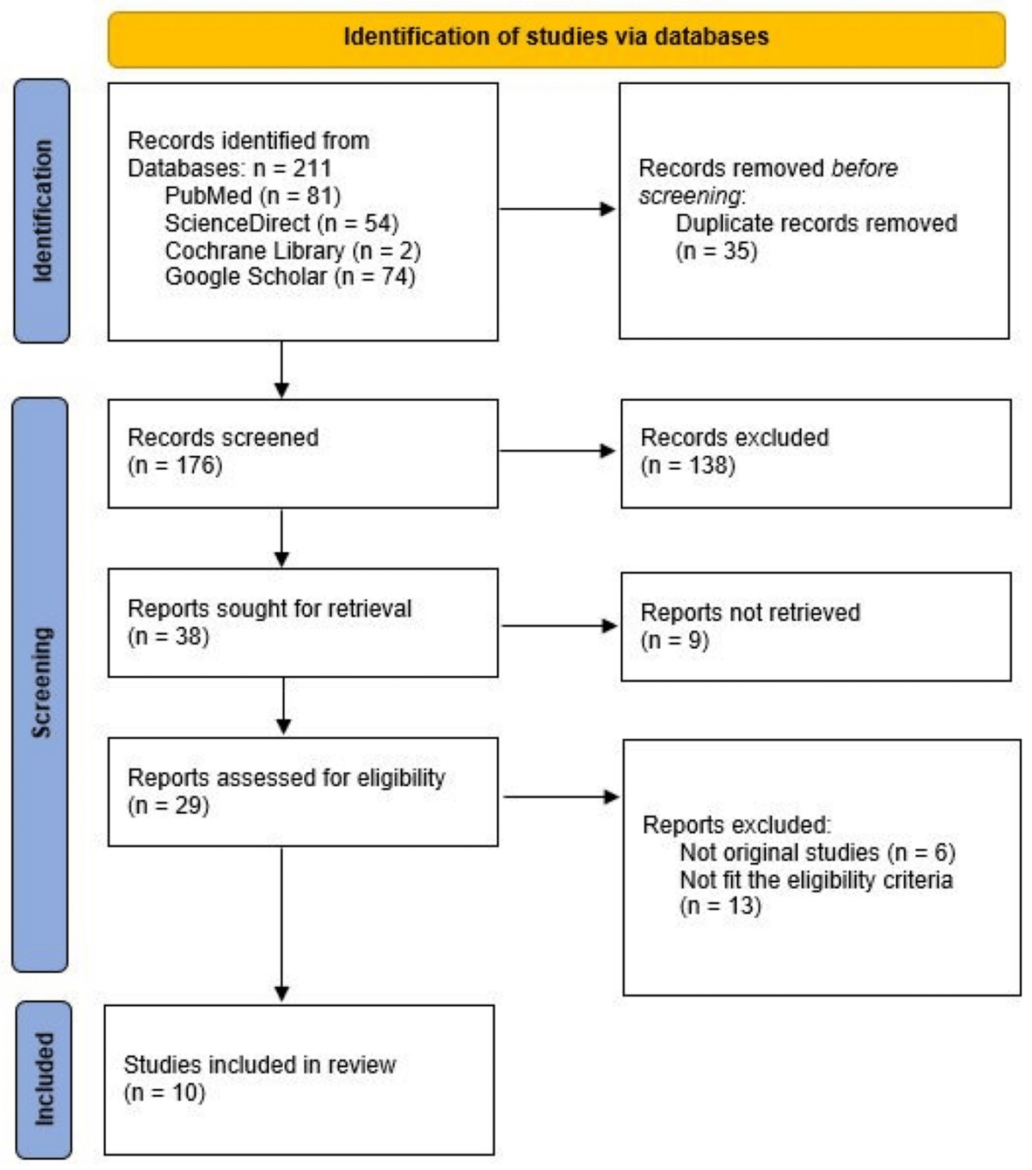
PRISMA flowchart showing the study selection process PRISMA: Preferred Reporting Items for Systematic Reviews and Meta-Analyses

All retrieved citations were imported into a reference management software (Zotero), and duplicates were removed. Two reviewers independently screened titles and abstracts for eligibility. Full-text articles of potentially relevant studies were then retrieved and assessed against the inclusion criteria. Disagreements between reviewers were resolved through discussion or consultation with a third reviewer. Studies categorized as “not retrieved” in the PRISMA flow diagram were excluded due to inaccessible full texts despite institutional access attempts. The study selection process was documented using a PRISMA flow diagram (Figure 1).

Data Extraction

Data were extracted independently by two reviewers using a standardized extraction form. Extracted information included study characteristics (authors, year, country, design), sample demographics, surgical details, aligner protocols, comparator information, follow-up durations, and outcomes.

Quality Assessment

The methodological quality and risk of bias of the included studies were evaluated using tools appropriate to each study design. Randomized controlled trials were assessed with the revised Cochrane Risk of Bias tool (RoB 2.0) [11]. For non-randomized studies, including cohort and case-control studies, the Newcastle-Ottawa Scale (NOS) was applied [12]. Case series were appraised using the Joanna Briggs Institute (JBI) critical appraisal checklist [13]. Two reviewers independently conducted the assessments, with any disagreements resolved through discussion or by consulting a third reviewer. 

Meta-Analysis

A meta-analysis was not conducted due to substantial heterogeneity among the included studies. The selected articles varied considerably in surgical procedures (single-jaw vs. bimaxillary, segmented vs. non-segmented osteotomies), timing and protocol of clear aligner use (surgery-first vs. orthodontics-first, differences in aligner systems, use of auxiliaries), and outcome measures (skeletal stability metrics, occlusal indices, periodontal assessments, and patient-reported outcomes). Follow-up durations also differed widely. These methodological inconsistencies precluded the pooling of data and prevented the generation of a meaningful quantitative synthesis; therefore, the findings were summarized through a narrative approach.

Data Synthesis

Findings were organized thematically around skeletal and dental stability, treatment efficiency, and patient-reported outcomes. Patterns, consistencies, and divergences across studies were systematically reviewed to provide a comprehensive interpretation of the current evidence.

Results

The electronic search yielded 211 records, of which 35 duplicates were removed before screening. A total of 176 titles and abstracts were reviewed, and 38 full-text articles were assessed for eligibility. After excluding studies that did not meet the inclusion criteria, ten studies were finalized for qualitative synthesis. The literature consisted of two RCTs [9,14], six retrospective comparative or case-control studies [15-20], and two retrospective case series [21,22]. In total, 258 patients were analyzed across the clinical studies. The two RCTs [9,14] were evaluated using the revised RoB 2.0 and demonstrated a low-to-moderate risk of bias (Table 2).

**Table 2:** Risk of Bias Assessment for Randomized Controlled Trials (RoB 2.0)

Study	Randomization Process	Deviations from Intended Interventions	Missing Outcome Data	Measurement of Outcomes	Selection of Reported Results	Overall Risk of Bias
Li et al. 2024 [12]	Low risk	Some concerns	Low risk	Low risk	Some concerns	Moderate
De Leyva et al. 2023 [7]	Low risk	Some concerns	Low risk	Low risk	Low risk	Low–Moderate

Six non-randomized comparative studies [15-20] were assessed using the NOS, and all were rated as moderate quality (Table 3).

**Table 3:** Quality assessment of non-randomized studies using the Newcastle-Ottawa scale (NOS)

Study	Selection (Max 4)	Comparability (Max 2)	Outcome/Exposure (Max 3)	Total Score (Max 9)	Quality
Meazzini et al. 2024 [14]	3	1	2	6/9	Moderate
Moon et al. 2021 [15]	3	1	2	6/9	Moderate
Meazzini et al. 2024 [16]	3	1	2	6/9	Moderate
Kankam et al. 2019 [17]	4	1	2	7/9	Moderate–Good
Kwon et al. 2022 [18]	3	1	2	6/9	Moderate
Cong et al. 2022 [13]	3	1	2	6/9	Moderate

Two case series [21,22] were appraised with the JBI critical appraisal checklist and were determined to be of moderate methodological quality (Table 4).

**Table 4:** Quality appraisal of case series using the JBI Critical Appraisal Checklist JBI: Joanna Briggs Institute

Study	Clear Inclusion Criteria	Consecutive Inclusion	Complete Inclusion	Clear Reporting of Demographics	Clear Reporting of Clinical Info	Valid Methods	Adequate Follow-Up	Reporting of Outcomes	Appropriate Statistical Analysis	Overall Quality
Nguyen et al. 2021 [19]	Yes	Yes	Yes	Yes	Yes	Yes	No	Yes	Not applicable	Moderate
Amodeo et al. 2020 [20]	Yes	Yes	Yes	Yes	Yes	Yes	No	Yes	Not applicable	Moderate

The basic characteristics of the included studies, including study design, sample size, surgical procedures, and post-surgical orthodontic protocols, are presented in Table 5.

**Table 5:** Summary characteristics of the included studies CAT: Clear Aligner Therapy; RCT: Randomized Controlled Trial; OFA: Orthodontics-First Approach; SFA: Surgery-First Approach; BSSO: Bilateral Sagittal Split Osteotomy; UCLP: Unilateral Cleft Lip and Palate; CAD/CAM: Computer-Aided Design/Computer-Aided Manufacturing; TADs: Temporary Anchorage Devices; OBAS: Orthodontic Bone Anchor Screws; PAR: Peer Assessment Rating

Study (Author, Year)	Country	Study Design	Sample Size (Clear Aligner/Fixed)	Population/Diagnosis	Surgical Procedure	Post-Surgical Orthodontic Protocol
Li et al. 2024 [12]	China	Randomized Controlled Trial (RCT)	74 (37/37)	Skeletal Class III Malocclusion	Bimaxillary (Le Fort I + BSSO)	SFA Group: Clear aligners started post-op. OFA Group: Pre-surgical decompensation with aligners, post-surgical finishing with aligners.
Meazzini et al. 2024 [14]	Italy	Retrospective Case-Control	26 (13/13)	Cleft Lip/Palate & Skeletal Class III with maxillary hypoplasia	Multi-segmental Le Fort I osteotomy (2- or 3-piece)	Pre-surgical decompensation with aligners. Bonded fixed appliances 1 month pre-op for surgical fixation; debonded at 3 months post-op to continue with aligner finishing.
Moon et al. 2021 [15]	South Korea	Preliminary Retrospective Comparative	15 (5/10)	Dentofacial deformities (Class II, Class III)	Mostly BSSO only; 1 patient with Bimaxillary (Le Fort I + BSSO)	Pre-surgical decompensation with aligners. Post-surgical elastic guidance protocol was the same for both groups.
Nguyen et al. 2021 [19]	Vietnam	Retrospective Case Series	13 (13/0)	Dentofacial deformities (Class II, Class III, Bimaxillary Protrusion, Asymmetry)	Bimaxillary (Le Fort I + BSSO) or Subapical Osteotomy	Surgery-First with Invisalign. Aligners started ~1 week pre-op and resumed ~1 week post-op. Used TADs for intermaxillary fixation/elastics.
Meazzini et al. 2024 [16]	Italy	Retrospective Case-Control	18 (9/9)	Unilateral Cleft Lip and Palate (UCLP) patients	2-piece Le Fort I osteotomy	Pre-surgical decompensation with aligners. Bonded fixed appliances pre-op for surgical fixation; debonded at 3 months post-op for aligner finishing.
Cong et al. 2022 [13]	USA, Canada, Brazil	Retrospective Cohort (Accuracy Study)	20 (20/0)	Orthognathic surgery patients (mostly Class III)	Not specified in detail	Pre-surgical decompensation entirely with Invisalign. Focused on the accuracy of this pre-surgical phase.
Amodeo et al. 2020 [20]	Italy	Case Series	12 (12/0)	Severe skeletal Class III malocclusion	Bimaxillary surgery (LeFort I + BSSO); some with genioplasty	Surgery-First approach with Invisalign. Aligners worn ~1 week pre-op and started 7-10 days post-op.
Kankam et al. 2019 [17]	USA	Retrospective Comparative	33 (13/20)	Dentofacial deformities requiring triple-jaw surgery	LeFort I, BSSO, and Genioplasty	Mixed protocols (likely pre-surgical ortho). Used orthodontic bone anchor screws (OBAS) for intermaxillary fixation.
De Leyva et al. 2023 [7]	Spain	Randomized Controlled Trial (RCT)	28 (14/14)	Dentofacial deformities (various)	Single-jaw or bimaxillary surgery (segmented & non-segmented)	Surgery-First approach. Invisalign group started aligners within first 10 days post-op.
Kwon et al. 2022 [18]	USA	Retrospective Cohort	15 (15/0)	Dentofacial deformities in AP, vertical, and transverse dimensions	Single or bimaxillary orthognathic surgery	Pre-surgical decompensation and post-surgical finishing with Invisalign. Used custom CAD/CAM splints (CAOS).

The applied orthodontic protocols fell into two main categories. The conventional Orthodontics-First Approach (OFA), as seen in studies such as Meazzini et al. [16] and Moon et al. [17], involved pre-surgical decompensation using clear aligners. Alternatively, the Surgery-First Approach (SFA) was implemented in studies such as Li et al. [14], Nguyen et al. [21], and Amodeo et al. [22], where orthognathic surgery was performed prior to any orthodontic treatment, with CAT initiated post-operatively. Detailed findings of included studies are summarized in Table 6.

**Table 6:** Outcomes, results, and findings of the included studies CAT: Clear Aligner Therapy; RCT: Randomized Controlled Trial; OFA: Orthodontics-First Approach; SFA: Surgery-First Approach; BSSO: Bilateral Sagittal Split Osteotomy; PAR: Peer Assessment Rating; OHIP-14: Oral Health Impact Profile-14; OQLQ-22: Orthognathic Quality of Life Questionnaire-22; NRS-10: Numeric Rating Scale-10; 3D: Three-Dimensional; CAD/CAM: Computer-Aided Design/Computer-Aided Manufacturing

Study (Author, Year)	Key Outcomes Assessed	Key Findings/Results	Conclusion
Li et al. 2024 [12]	Skeletal stability (6 months post-op); overall treatment duration	No significant difference in skeletal stability between SFA+CAT and OFA+CAT groups, except in maxillary yaw; the SFA+CAT group had significantly shorter overall treatment time (18.05 vs. 22.83 months, p<0.05)	The combination of SFA and clear aligners achieves comparable skeletal stability to the conventional approach while significantly reducing total treatment time.
Meazzini et al. 2024 [14]	Number of pre-surgical refinements/impressions; pre-surgical treatment time; number of appointments	Fewer refinements needed with aligners (0.4±0.4 vs. 1.8±0.6 impressions, p>0.05); similar pre-surgical treatment time (18.4 vs. 16.3 months, p>0.05); significantly fewer appointments with aligners (5.1 vs. 10.2, p<0.05)	Clear aligners are effective for pre-surgical preparation in complex multi-segmental cases when aided by a simple superimposition technique, offering the benefit of fewer clinical appointments.
Moon et al. 2021 [15]	Postoperative skeletal stability (6 months); pre-surgical orthodontic period; number of extractions	No significant difference in 6-month postoperative stability between groups; shorter pre-op time and fewer extractions in the aligner group, but not statistically significant	Orthognathic surgery with clear aligners is feasible and shows similar short-term stability to fixed appliances. The aligner group showed a trend towards less complex pre-surgical treatment.
Nguyen et al. 2021 [19]	Treatment feasibility & workflow; patient satisfaction & esthetic outcomes; occlusal and skeletal results	Successful treatment of various complex deformities using a fully digital Surgery-First protocol; good occlusal and esthetic results achieved in all patients; high patient satisfaction reported	The combination of advanced digital 3D planning, Surgery-First approach, and CAT is a viable and effective novel protocol that enhances patient quality of life.
Meazzini et al. 2024 [16]	Adequacy of pre-surgical preparation (qualitative); treatment duration & appointments	Aligners achieved acceptable pre-surgical coordination with 1 or no refinements; similar treatment duration to fixed appliances; confirmed significantly fewer appointments with aligners	The proposed superimposition method makes clear aligners a viable and less appointment-intensive alternative for pre-surgical preparation in cleft patients requiring segmental osteotomies.
Cong et al. 2022 [13]	Accuracy of predicted vs. achieved tooth movements (angular & linear)	Average accuracy of 63.4% ± 11.5% for pre-surgical movements; linear movements more accurate than angular; movements for arch leveling and decompensation showed relatively high accuracy	Invisalign is a valid modality for pre-surgical orthodontics, as the movements required for decompensation and leveling can be achieved with clinically acceptable accuracy.
Amodeo et al. 2020 [20]	Dental & skeletal stability; occlusion (angle class); treatment duration; complications	Achieved stable Angle Class I occlusion at 1-year follow-up; no surgical-orthodontic relapse reported; average orthodontic treatment time: 10 months	The combination of Surgery-First and Invisalign is a valid protocol for managing Class III malocclusion, offering good stability, aesthetics, and patient comfort.
Kankam et al. 2019 [17]	Perioperative outcomes (OR time, hospital stay, etc.; postoperative edema (3D volumetric); complications	No significant differences in OR time, hospital stay, diet, or narcotic use between groups; post-op edema was similar (Clear Aligner: 37.36 cm³ vs. Fixed: 44.29 cm³; p=0.712)	Complex orthognathic surgery can be successfully performed with clear aligners without compromising perioperative outcomes or increasing post-op edema.
De Leyva et al. 2023 [7]	Periodontal health (Plaque Index, Bleeding on Probing, Probing Depth); Quality of Life (OHIP-14, OQLQ-22); Pain (NRS-10); Treatment duration	Significantly better periodontal health in the Invisalign group at final assessment (all p<0.05); significantly better quality of life scores in the Invisalign group (OHIP-14 p=0.004; OQLQ-22 p=0.002); treatment duration was similar between groups (p=0.575)	In a surgery-first protocol, clear aligners lead to significantly better periodontal health and patient-reported quality of life compared to fixed appliances.
Kwon et al. 2022 [18]	Occlusal Outcome (Weighted PAR Index)	87% improvement in PAR score (Pre: 27.63 ±12.09 vs. Post: 3.5 ±2.54; p=0.001); all PAR subcategories improved significantly except for midline	Orthognathic surgery with clear aligners can achieve a high standard of occlusal outcome, as measured by the PAR index. The results are comparable to those reported with fixed appliances.

Clinical Efficacy and Treatment Efficiency

Regarding clinical outcomes, the included studies demonstrated that CAT is a clinically effective modality. The skeletal stability achieved with CAT was found to be comparable to that of fixed appliances. The RCT by Li et al. [14] reported no significant differences in skeletal relapse at six months post-operatively between the SFA+CAT and OFA+CAT groups. Similarly, Moon et al. [17] found no significant difference in short-term postoperative stability. Occlusal outcomes were also favorable; Kwon et al. [20] demonstrated an 87% improvement in the Peer Assessment Rating (PAR) index, and Amodeo et al. [22] reported achieving stable Angle Class I occlusion. In terms of efficiency, Li et al. [14] reported a significantly shorter overall treatment time for the SFA+CAT group. Furthermore, Meazzini et al. [18] consistently found that the use of CAT for pre-surgical preparation was associated with a significant reduction in the number of required clinical appointments.

Patient-Centered Outcomes and Current Adoption

From a patient-centered perspective, clear aligners offered distinct advantages. The RCT by de Leyva et al. [9] provided high-quality evidence that patients treated with CAT in a surgery-first protocol experienced significantly better periodontal health and superior quality of life scores compared to those in fixed appliances. High levels of patient satisfaction were also reported in case series such as Nguyen et al. (2021) [21]. From a surgical standpoint, Kankam et al. (2019) [19] confirmed that performing orthognathic surgery with CAT did not compromise perioperative outcomes.

Discussion

In this systematic review, we evaluated the use of CAT in the context of orthognathic surgery (pre- and/or post-surgical phases), focusing on three core domains: (1) treatment efficiency and duration, (2) post-treatment stability (skeletal, dental and occlusal), and (3) patient-centred outcomes (comfort, hygiene, esthetics, satisfaction). The available evidence, albeit limited, suggests that clear aligners can be a viable alternative to fixed appliances in surgical orthodontics under certain conditions-though important caveats remain.

Several of the included studies [14,16] reported a reduction or at least no prolongation of overall treatment time when clear aligners were used in a surgical workflow. This finding aligns with earlier work in non-surgical orthodontics: one systematic review found that clear aligners showed shorter chair-time and treatment duration in mild-to-moderate cases compared with fixed appliances, although no difference in stability or occlusion was found [23]. In the surgical context, the streamlined digital workflow (3D setups, intraoral scanning, aligner manufacturing) appears to facilitate predictable movements and may reduce ancillary visits (e.g., for wire adjustments) compared to conventional brackets [23-25].

However, caution is warranted because many of the included studies had small sample sizes and heterogeneous protocols, including variations in aligner systems, use of auxiliary attachments, and surgical sequencing (surgery-first vs. orthodontics-first). For example, Nguyen et al. [21] and Amodeo et al. [22] emphasized the importance of combining aligners with skeletal anchorage and precise digital planning to ensure predictable movements, while Kwon et al. [20] highlighted protocol-dependent variability in occlusal finishing. These methodological differences limit the generalizability of conclusions. Moreover, as noted by Jaber et al. , CAT in complex or surgical cases still lacks robust randomized data and long-term follow-up [24]. Therefore, while aligners show promise for improved efficiency, universal reductions in treatment duration should not be assumed across all surgical orthodontic cases.

A major clinical concern in surgical orthodontics is post-treatment skeletal and occlusal stability. In this review, most studies-including Li et al. [14], Moon et al. [17], and Kwon et al. [20]-reported comparable short- to mid-term stability between aligner-treated and fixed-appliance patients. Specifically, Li et al. [14] found no significant difference in skeletal relapse at six months between surgery-first and orthodontics-first aligner groups, while Moon et al. [17] observed similar short-term stability at six months post-surgery. Kwon et al. [20] documented an 87% improvement in the PAR index, confirming satisfactory occlusal finishing comparable to conventional methods. These findings collectively suggest that clear aligners can maintain skeletal and dental outcomes in orthognathic cases.

In the broader orthodontic literature (non-surgical), aligners and fixed appliances have been shown to have similar post‐treatment stability in many cases - but with caveats. For instance, the meta‐analysis by Caruso et al. reported no significant difference in stability between aligners and fixed appliances in non-extraction cases, yet in extraction cases, fixed appliances provided better control and perhaps better long-term stability [26]. In the surgical arena, there is added complexity: skeletal relapse risk (especially Class III mandibular growth), neuromuscular adaptation, and soft-tissue influences. The scoping review by Lugas et al. noted these as potential risks when using aligners in orthognathic workflows [1].

Among the included studies, Li et al. [14] reported comparable skeletal stability between aligner-based and conventional groups, while Kwon et al. [20] demonstrated excellent occlusal outcomes with significant PAR score improvements. Nevertheless, heterogeneity remains evident. Moon et al. [17] and Meazzini et al. [18] differed in their pre-surgical and post-surgical aligner usage protocols, as well as the degree of surgical movement, making direct comparisons difficult. Accordingly, clinicians should prioritize robust retention strategies and encourage consistent post-treatment follow-up beyond the conventional 6- to 12-month period. Evidence from non-surgical studies has shown measurable relapse in anterior alignment after three years among aligner patients [24], underscoring the need for long-term monitoring in surgical cohorts.

From a patient-centered perspective, clear aligners demonstrated distinct advantages in comfort, esthetics, and periodontal health. The RCT by de Leyva et al. [9] found that aligner-treated patients reported significantly better oral health-related quality of life (OHIP-14 and OQLQ-22) and periodontal indices compared with those treated using fixed appliances. Similar observations were made by Nguyen et al. [21], who reported high satisfaction scores and favorable esthetic outcomes following a fully digital, surgery-first protocol integrating clear aligners. These findings align with broader orthodontic evidence that aligner systems are associated with fewer white-spot lesions, easier oral hygiene maintenance, and reduced soft-tissue irritation compared to fixed brackets [2,3]. However, these benefits are dependent on patient compliance. Studies suggest that only 45-50% of aligner users adhere to the recommended ≥22 hours/day wear schedule [23]. In surgical orthodontic cases, poor compliance could compromise occlusal refinement or prolong treatment, particularly when intermaxillary elastics or aligner refinements are required.

Despite the promising findings of this review, several important limitations must be acknowledged. First, the body of evidence is predominantly comprised of small-scale, retrospective case series and observational studies, which limit statistical power and increase susceptibility to selection and reporting bias. The heterogeneity of treatment protocols - including the use of different aligner systems, variable auxiliary mechanics, differing surgical procedures, and inconsistent retention strategies - also impedes meaningful cross-study comparisons and meta-analytic synthesis. Second, the majority of studies present only short- to mid-term outcomes (e.g., 6-12 months follow-up), meaning that long-term stability, relapse rates, and retention effects in the context of combined surgical-aligner workflows remain under-documented. Third, patient-reported outcomes, such as quality of life, satisfaction, and functional metrics, are inconsistently reported, which limits comprehensive evaluation of the real-world clinical value from the patient perspective. Fourth, because removable aligner treatment is highly dependent on patient compliance (wear time, maintenance, follow-through with protocols), variation in adherence may confound outcomes, yet many included studies did not quantify or adjust for compliance. Although study screening and data extraction were performed independently by two reviewers with consensus resolution of discrepancies, formal inter-rater reliability statistics were not calculated, which may limit the quantitative assessment of reviewer agreement. Finally, the absence of large RCTs directly comparing aligner-based versus fixed-appliance orthodontic management in surgical patients means that causality remains tentative and recommendations must be interpreted cautiously. Together, these limitations highlight the need for well-designed prospective trials, standardized protocols, and longer-term follow-up to confirm the initial encouraging results of aligner use in orthognathic workflows.

## Conclusions

This systematic review demonstrates that CAT is a viable and effective modality for post-surgical orthodontic management in patients undergoing orthognathic surgery. Across the included studies, clear aligners provided skeletal and dental stability comparable to traditional fixed appliances, with no significant differences in short-term relapse. Several studies reported potential advantages in treatment efficiency, including reduced overall treatment duration in selected surgery-first protocols and fewer clinical appointments during pre-surgical preparation; however, these findings were derived from studies with variable and often limited sample sizes and heterogeneous methodologies. Similarly, although patient-centered outcomes such as comfort, periodontal health, and quality of life tended to favor clear aligners in individual studies, these observations should be interpreted cautiously and cannot be generalized across all orthognathic populations. Evidence from RCTs showed significantly better periodontal health, greater comfort, and improved quality of life compared with fixed appliances. Moreover, clear aligner-based protocols did not negatively affect perioperative parameters, demonstrating that their use is safe in complex surgical workflows. Despite these promising findings, the current evidence base remains limited by heterogeneity in surgical procedures, aligner protocols, and outcome measures, as well as a scarcity of long-term controlled studies. High-quality, standardized clinical trials with extended follow-up are needed to confirm the long-term stability of clear aligner-assisted surgical outcomes and to refine protocol guidelines. Overall, clear aligners represent an effective, patient-friendly alternative to fixed appliances in post-surgical orthodontic care, offering comparable clinical outcomes with potential benefits in comfort, periodontal health, and treatment efficiency. As digital planning and surgical-orthodontic integration continue to advance, clear aligners are likely to play an increasingly significant role in contemporary orthognathic treatment pathways.

## Appendices

Table 7 presents the search strategies for the included studies.

**Table 7:** Search strategies for the included studies Total records identified: 211; duplicates removed: 35

Database/Register	Search String	Records Retrieved
PubMed/MEDLINE	("clear aligner" OR "clear aligner therapy" OR "Invisalign" OR "clear-aligner") AND ("orthognathic surgery" OR "jaw surgery" OR "dentoalveolar surgery" OR "surgery-first" OR "orthodontics-first" OR "post-surgical orthodontics") AND ("stability" OR "skeletal stability" OR "dental stability" OR "treatment efficiency" OR "occlusion" OR "periodontal health" OR "quality of life" OR "patient-reported outcomes")	81
ScienceDirect	("clear aligner" OR "Invisalign") AND ("orthognathic surgery" OR "jaw surgery" OR "post-surgical orthodontics") AND ("stability" OR "treatment outcomes" OR "efficiency")	54
Cochrane Library	("clear aligner" OR "Invisalign") AND ("orthognathic surgery" OR "jaw surgery" OR "postoperative orthodontics")	2
Google Scholar	"clear aligner" AND "orthognathic surgery" OR "post-surgical orthodontics"	74
